# Preconception and Prenatal Environment and Growth Faltering Among Children in Uganda

**DOI:** 10.1001/jamanetworkopen.2025.1122

**Published:** 2025-03-19

**Authors:** Paddy Ssentongo, Claudio Fronterre, Jessica E. Ericson, Ming Wang, Laila Al-Shaar, Helen Greatrex, Philip O. Omadi, Joseph Muvawala, Steven J. Greybush, Pamela K. Mbabazi, Laura E. Murray-Kolb, Abraham J. B. Muwanguzi, Steven J. Schiff

**Affiliations:** 1 Division of Infectious Diseases and Epidemiology, Department of Medicine, Penn State Hershey Medical Center, Hershey, Pennsylvania; 2 Department of Public Health Sciences, The Pennsylvania State University College of Medicine, Hershey; 3 Centre for Health Informatics, Computing and Statistics, Lancaster University, Lancaster, United Kingdom; 4 Department of Pediatrics, Penn State Hershey Medical Center, Hershey, Pennsylvania; 5 Department of Population and Quantitative Health Sciences, Case Western Reserve University, Cleveland, Ohio; 6 Department of Meteorology and Atmospheric Science, The Pennsylvania State University, University Park; 7 National Planning Authority, Kampala, Uganda; 8 Department of Nutrition Science, College of Health and Human Sciences, Purdue University, West Lafayette, Indiana; 9 Ministry of Science, Innovation and Technology, Kampala, Uganda; 10 Department of Neurosurgery, Yale University, New Haven, Connecticut; 11 Institute for Computational and Data Sciences, The Pennsylvania State University, University Park; 12 Department of Epidemiology of Microbial Diseases, Yale University, New Haven, Connecticut

## Abstract

**Importance:**

Children with growth faltering are more susceptible to infections and may experience cognitive, physical, and metabolic developmental impairments.

**Objective:**

To assess whether prenatal and preconception meteorological and environmental factors are associated with village-level rates of childhood growth outcomes in Uganda.

**Design, Setting, and Participants:**

This cross-sectional study used data collected between June 20, 2015, and December 16, 2016, from the 2016 Ugandan Demographic and Health Survey for individuals aged 0 to 59 months with available anthropometric measures (weight and length or height). Data analysis was conducted from October 2020 to April 2024.

**Exposures:**

Factors assessed included meteorological information, such as drought index (Standardized Precipitation-Evapotranspiration Index [SPEI]), Aridity Index, rainfall, temperature, and vegetation indices; demographic and economic development factors (nighttime light emissions, driving time to the nearest city); and land topography (slope angle, elevation above sea level).

**Main Outcomes and Measures:**

The main outcomes were height-for-age *z* score (HAZ), weight-for-age *z* score (WAZ), and weight-for-height *z* score (WHZ). Spatial resolution estimates, at 1 km × 1 km of childhood growth faltering indicators, were created.

**Results:**

Of the 5219 individuals aged 0 to 59 months included in the analysis, 2633 (50%) were female; mean (SD) age was 29 (17) months. Of these individuals, 30.22% (95% CI, 29.36%-30.98%) had stunting, 12.23% (95% CI, 11.55%-12.91%) had underweight, and 3.63% (95% CI, 3.46%-3.80%) had wasting. Large disparities in the burden of childhood growth faltering existed within Uganda at smaller and larger spatial scales; villages in the northeastern and southwestern areas of the country had the highest prevalence of all forms of growth faltering (stunting, >40%; underweight, >16%; and wasting, >6%). Higher SPEI at 3 months before birth was positively associated with all childhood growth outcomes: HAZ (β, 0.06; 95% CI, 0.02-0.10), WAZ (β, 0.04; 95% CI, 0.01-0.07), and WHZ (β, 0.03; 95% CI, 0.001-0.06). Higher location mean rainfall 11 months before birth was also positively associated with HAZ (β, 0.06; 95% CI, 0.01-0.10). Aridity Index associations with WAZ (β, 0.09; 95% CI, 0.04-0.13) and WHZ (β, 0.09; 95% CI, 0.02-0.16) were consistent with findings for SPEI.

**Conclusions and Relevance:**

In this study of 5219 individuals 0 to 59 months of age in Uganda, rainfall and long-term availability of water at preconception and during gestation were positively associated with nutritional child growth outcomes. Understanding the relative contributions of meteorological environment factors on the spatial distribution of undernutrition at various spatial scales within Uganda (from the village to the district level) may help in the design of more cost-effective delivery of precision public health programs.

## Key Points

**Question:**

Are preconception and prenatal meteorological and environmental factors associated with the risk of postnatal childhood growth faltering?

**Findings:**

In this cross-sectional study of 5219 individuals aged 0 to 59 months in Uganda, precipitation and water availability before conception and birth were associated with later childhood growth faltering.

**Meaning:**

The findings suggest that rainfall and water availability in the preconception and prenatal periods may be associated with childhood growth outcomes.

## Teaser

This cross-sectional study examines whether meteorological and environmental factors before conception and during gestation are associated with stunting, underweight, and wasting among individuals younger than 5 years in Uganda.

## Introduction

Growth faltering is defined by stunting, a failure to achieve age-related metrics of normal length or height, and wasting, a substantially reduced weight for length or height. Although the deaths associated with childhood stunting and wasting have been decreasing globally, they remain estimated to be above 257 000 and 1 100 000 yearly, respectively.^1^ Both stunting and wasting are associated with decreased neurocognitive development.^2^

It has recently been shown that growth faltering most often begins at or shortly after birth. Although the prevalence of linear growth faltering as stunting peaked at about 18 months of age in 1 study,^3^ in another study, the highest incidence of stunting occurred from birth to 3 months of age, and incident stunting was highest at birth.^4^ Maternal height, weight, and body mass index were associated with the greatest risk of stunting at birth and in future linear growth trajectories. The majority of children (86%) who ever had stunting had length for age less than the median at birth. Similarly, wasting prevalence was highest at birth.^5^ Early wasting is associated with increased risk of later growth faltering, and wasting, unlike stunting in isolation, is associated with increased risk of mortality.^5^

Stunting is a long-term response to a sustained poor dietary intake or repeated illnesses.^6^ Wasting is a short-term (acute) manifestation of undernutrition in response to an inadequate intake of calories and/or an infectious process.^7^ Compared with stunting and underweight, wasting is more sensitive to external environmental fluctuations,^7^ with an increase in the infectious disease burden after birth during rainy seasons,^5^ and has greater consequences for children over months rather than over years. Wasting, unlike stunting, is an acute measure of malnutrition and can lead to recovery. Nevertheless, early wasting is also associated with an increased risk of stunting and poor neurocognitive development.^5^ In addition, wasting has been associated with increased rainfall after birth, with recovery seen in the dry seasons following rains.^5^ Wasting is also associated with a weakened immune system, leading to an increased risk of infections.^5^ Across many countries, weight-for-length *z* score can be at a minimum during rainy seasons and increases are observed during dry seasons.^5^ Furthermore, weight-for-length *z* score at birth is dependent on the month of birth but counterintuitively reaches an average minimum just before or at the beginning of a rainy season.^5^ In addition to food insecurity, rainy seasons are associated with an increased risk of infectious diseases, such as malaria^8,9^; bacterial infections; and postinfectious hydrocephalus.^10^ The complex relationship between rainfall and wasting remains incompletely studied at present.

If growth faltering is present at birth and affected by environmental factors, such as rainfall, preconceptual and prenatal environmental factors may be associated with this early growth faltering. We aimed to investigate whether such associations could be identified in Uganda.

## Methods

### Overview

In this cross-sectional study, we hypothesized that childhood growth faltering risk might be associated with (1) the long-term water availability at an individual’s location, characterized by the Standardized Precipitation-Evapotranspiration Index (SPEI) and the Aridity Index; (2) rainfall and temperature variations that affect infectious diseases, agricultural activities, and crop diversity and yield; and (3) topography that affects water runoff, soil erosion, soil fertility, and mineral concentration. We also examined measures of vegetation and demographic and economic development as possible factors. The Uganda Demographic and Health Survey (UDHS) protocols and guidelines were reviewed and approved by the Ugandan Ministry of Health (MOH) ethical review committee and the institutional review board (IRB) of ICF International, USA. The study’s research and methods were performed per the regulations and guidelines of the Ugandan MOH ethical review committee and the IRB of ICF International, USA. Written informed consent was obtained from the parents or guardians of each participant before the survey. The UDHS program permitted use of the data. All data were anonymous; therefore, we did not seek further ethical clearance. We followed the Strengthening the Reporting of Observational Studies in Epidemiology (STROBE) reporting guideline.^11^

Measurement and assessment of country-level spatial variations in the prevalence of childhood growth faltering is essential for international comparisons but masks disparities in childhood growth faltering at the subnational level, where targeted resource allocation and interventions can be implemented for populations with the greatest need.^12,13^ Geostatistical models were therefore parameterized to generate childhood growth faltering spatial risk surfaces at 1 km × 1 km grid cell levels and to quantify the absolute case numbers of individuals with growth faltering. Absolute numbers of individuals with growth faltering were derived using the 2020 population younger than 5 years in Uganda, with the assumption that the prevalence of growth faltering did not change substantially between 2016 and 2020.

To explore associations, we lagged the time-varying meteorological factors from month of birth to 12 months prior to birth. Our primary objective was to characterize meteorological, environmental, and sociodemographic factors prior to birth that are associated with childhood growth faltering after birth. The secondary objective was to estimate the number of individuals younger than 5 years with growth faltering at a granular spatial scale to guide precision public health interventions.

### Data Sources and Participants

Data were extracted from the 2016 UDHS.^14^ The data were collected between June 20, 2015, and December 16, 2016, by the Uganda Bureau of Statistics in collaboration with the Uganda Ministry of Health and coordinated by the Demographic and Health Survey Program in Maryland. The details of sampling methods are discussed elsewhere.^14^ In summary, data collection involved a multistage stratified sampling design. First, Uganda was divided into 15 regions. Within these regions, populations were stratified by urban and rural areas of residence. Within these stratified areas, 696 clusters were randomly selected. Clusters were selected based on a probability proportional to the population size. In Uganda, a cluster is a geographic area that covers an average of 130 households. During the second sampling stage, all households within a cluster were listed from the most recent population census (2014), and 30 households per cluster were randomly sampled. In total, a representative sample of 20 880 households were selected. Length or height and weight information was collected from eligible individuals aged 0 to 59 months.

### Assessment of Childhood Growth Faltering Status

We analyzed the data for stunting, underweight, and wasting in individuals aged 0 to 59 months at the time of the interview. Those aged 24 months or younger had their length measured in recumbent position, and those older than 24 months had height measured while standing. Length was measured with a ShorrBoard measuring board (Weight and Measure, LLC). Height was measured with an electronic seca 878 flat scale (seca GmbH) (accuracy, 0-50 kg ± 50 g or 50-150 kg ± 100 g). The *z* scores for weight for age, length or height for age, and weight for length or height were provided in the UDHS data and calculated using the 2006 World Health Organization (WHO) Child Growth Standards (height-for-age *z* score [HAZ], weight-for-age *z* score [WAZ], and weight-for-height *z* score [WHZ]).^15^ An individual was considered to have stunting, underweight, or wasting if they exhibited a *z* score below −2 SDs from the reference population norm.

### Meteorological and Environmental Data

Our models were parameterized by high-resolution gridded meteorological data at 30 arc seconds (1 km at the equator). The covariates were extracted from the gridded raster cells using the global positioning system location of each individual. Details of data sources are provided in eTable 1 in Supplement 1. These included rainfall, land surface mean temperature, Aridity Index, SPEI, and Enhanced Vegetation Index (EVI) or Normalized Difference Vegetation Index (NDVI) in the individual’s location and were lagged from the month of birth to 12 months before birth. Datasets obtained at a coarser spatial scale were regridded (interpolated from a larger grid resolution to a 1 km ×1 km spatial scale). For the mean monthly rainfall, we calculated the mean daily observed values for each month from January 1, 2011, to December 31, 2016, at each location. For the land surface mean temperature, we calculated the mean monthly observed values for each year from January 1, 2011, to December 31, 2016, at a 1 km ×1 km spatial scale.

### Drought Index

We used the SPEI, a region-specific indicator of deviations from the average long-term water balance (precipitation minus potential evapotranspiration) (eFigure 1 in Supplement 1).^16^ By accounting for water lost to evapotranspiration, the SPEI can more accurately indicate the overall water availability and agricultural stress at a specific geographic location. Furthermore, because this metric is based on long-term norms for a given location, it characterizes precipitation extremes in a comparable way among locations. SPEI is multiscalar, with timescales between 1 and 48 months and *n* representing the cumulative water balance over the previous *n* months. To choose an appropriate timescale at which water balance variations were associated with vegetation activity, we first estimated the correlation of vegetation indices with the SPEI. Previous global analysis showed that varying timescales of the SPEI showed varying degrees of correlation with vegetation indices.^17^ In the present analysis, a 2-month SPEI estimate demonstrated the highest Pearson correlation coefficient compared with other timescales (eFigure 2 in Supplement 1). In our modeling approach, the association between increasing lags of 2-month SPEI values and child nutritional outcome was explored.

Although the Aridity Index and SPEI incorporate precipitation and evapotranspiration (effect of temperature) in their derivation, their impact on agriculture is different because the Aridity Index is derived from mean annual rainfall (long-term effects); however, a shorter temporal resolution of mean monthly precipitation is used to estimate SPEI.^18^ Given that Uganda’s stable diet is based on crops with short growing cycles (eg, beans, nuts, and potatoes), SPEI has a greater correlation than the chronic long-term influence of the Aridity Index.^19,20^ Nevertheless, the association of these 2 metrices of water balance with annual food crops (eg, bananas) is likely to be the same.

### Mean Monthly Rainfall

Rainfall data (in millimeters) were obtained from the African Rainfall Estimation Algorithm, version 2 (RFE 2.0).^21^ Rainfall data provided are daily gridded, satellite-based rainfall estimates from the National Oceanic and Atmospheric Administration produced at a spatial resolution of 0.1° × 0.1°. RFE 2.0 combines satellite thermal infrared data with passive microwave data and Global Telecommunication System rain gauge data.^22^ RFE 2.0 is open source and compares well with other rainfall products for continental Africa.^23^ We calculated the mean monthly rainfall by calculating the mean daily and location-specific observed values from January 1, 2011, to December 31, 2016 (eFigure 3 in Supplement 1).

### Land Surface Mean Temperature

Monthly, 0.5° spatial resolution mean temperature data were obtained from gridded Climatic Research Unit Time Series (CRU TS).^24^ Data are derived by the interpolation of monthly climate anomalies from extensive networks of weather station observations. We used CRU TS, version 4, which spans from 1901 to 2019. For modeling purposes, we used the average temperature from 2011 to 2016 interpolated to 1-km spatial resolution (eFigure 4 in Supplement 1).

### NDVI and EVI

The NDVI and EVI values were produced using data collected by the Moderate Resolution Imaging Spectroradiometer aboard the National Aeronautics and Space Administration’s Terra satellite (MOD13C1 and MOD13C2). These NDVI data are robust, empirical measures of vegetation activity at the land surface. NDVI can be affected by the topography of the land surface.^25^ Therefore, EVI was created with a correction factor to account for possible distortion. We first compared monthly NDVI and EVI values. Because these 2 products were highly correlated (*r* = 0.98; *P* < .001) (eFigure 5 in Supplement 1), EVI was selected for use in the final model (eFigure 6 in Supplement 1).

### Aridity Index

To account for the long-term water availability at an individual’s location, we used the Global Aridity Index, which provides high-resolution (30 arc seconds) raster climate data from 1970 to 2000.^26^ The Aridity Index is the ratio between mean annual precipitation and mean annual reference evapotranspiration based on the implementation of the Penman-Monteith evapotranspiration equation for reference crops and using WorldClim, version 2.0 data.^27^ The Aridity Index indicates rainfall over potential vegetation water demand (aggregated on an annual basis), and its value thus increases under more humid conditions and decreases with more arid conditions (eFigure 7 in Supplement 1).

### Demographic and Economic Development Factors

To determine the absolute counts of individuals with undernutrition for each grid cell, we incorporated the 2020 population count of individuals younger than 5 years within the corresponding grid cell. We used nighttime light emissions as a proxy for human development and the degree of urbanization (eFigure 8 in Supplement 1).^28^ Furthermore, we explored driving time to the nearest city (settlements with a population >50 000) as a proxy for expanding road networks, access to opportunities to work, and health care (eFigure 9 in Supplement 1).^29^

### Slope Angle and Elevation Above Sea Level

Elevation above sea level and the slope angle are additional determinants of crop yield. The degree of slope angle can affect water runoff and soil erosion, which can affect agricultural productivity. Runoff increases at steeper slopes.^30^ Additionally, altitude is a determinant of neighborhoods. In the urban regions of Uganda, living in elevated areas is preferred, and these areas tend to be populated by individuals with higher socioeconomic status. However, in rural locations, living in mountainous regions negatively impacts access to health care and increases the slope gradient and soil erosion, all of which can be associated with worse nutritional status among mothers and their offspring.^31^ In our analysis, elevation was highly correlated with the slope angle (eFigure 10 in Supplement 1). We chose to include slope angle in the modeling due to its impact on agricultural practices (eFigure 11 in Supplement 1).

### Statistical Analysis

The modeling framework used to generate fine-scale risk maps for undernutrition in Uganda is based on a body of statistical theory known as model-based geostatistics.^32^ These methods are a class of generalized linear mixed models characterized by spatially correlated random effects that capture residual spatial variation present in the data. While it is common to first classify a child as malnourished or not based on the *z* scores and then model their malnutrition status, it has been shown that this dichotomization process leads to loss of information.^33^ For this reason, we modeled the *z* scores directly and then calculated the prevalence of stunting, underweight, and wasting using the following estimated distribution.

We let *Y_ij_* denote the HAZ, WAZ, and WHZ of the *j*th individual at cluster location *x_i_*. We defined the linear geostatistical model as follows: *Y_ij_* = α + β*^T^D*(*x_i_*) + *S*(*x_i_*) + *U_ij_*: *i* = 1, …, *n*, where α is the model intercept, *D*(*x_i_*) is a vector of explanatory variables at location *x_i_* with associated regression coefficients β, *S*(*x_i_*) is a spatial gaussian process, and the *U_ij_* values are independent and normally distributed random effects with a mean of 0 and variance *w*^2^ representing individual-specific residual variation at location *x_i_*.

We modeled *S*(*x*) as a stationary and isotropic gaussian process with a mean of 0, variance σ^2^, and correlation function Cor{*S*(*x*), *S*(*x'*)} = *p*(*u*), where *u* = ||*x* − *x'||* denotes the Euclidean distance between *x* and *x'*. We adopted an exponential correlation function, *p*(*u*) = exp{−*u*/ϕ}, with ϕ as a scale parameter that indicates the strength of the spatial correlation (ie, a larger value of ϕ indicates that spatial correlation persists over a longer distance). For the exponential correlation function above the practical range, the distance at which *p*(*u*) = 0.05 is approximately equal to 3ϕ. If there is no evidence of residual spatial correlation as assessed by the inspection of the variogram, *p*(*u*) = 0 and *S*(*x*) become another set of independent random effects that represent residual nonspatial variation at the cluster level.

We tested which lags for monthly EVI, rainfall, and SPEI better described the observed distribution of childhood growth outcomes in Uganda, considering lags *d* = 1, …, *D*, where *D* = 12 months (a whole year) before birth. Each lag was tested on its own. Because the lag effects are highly correlated, each lag was tested independently for each factor to minimize spurious relationships.

Monte Carlo maximum likelihood was used both for parameter estimation and to obtain the full joint estimated distribution of HAZ, WAZ, and WHZ at each location on a regular 1 km ×1 km spatial grid overlaying Uganda.^34,35^ The inference and the estimation stages were executed using the prevmap package in R, version 4.2.0 (R Project for Statistical Computing).^36^ To provide estimates at the district administrative level, where program planning and decision-making occur, we aggregated undernutrition prevalence and the absolute counts at the district level using the underlying gridded population. Map values were written to a raster that can be downloaded for operational purposes. Data were analyzed from October 2020 to April 2024. Two-sided *P* < .05 was considered significant.

## Results

### Overall Prevalence of Stunting, Wasting, and Underweight

**Figure 1.**
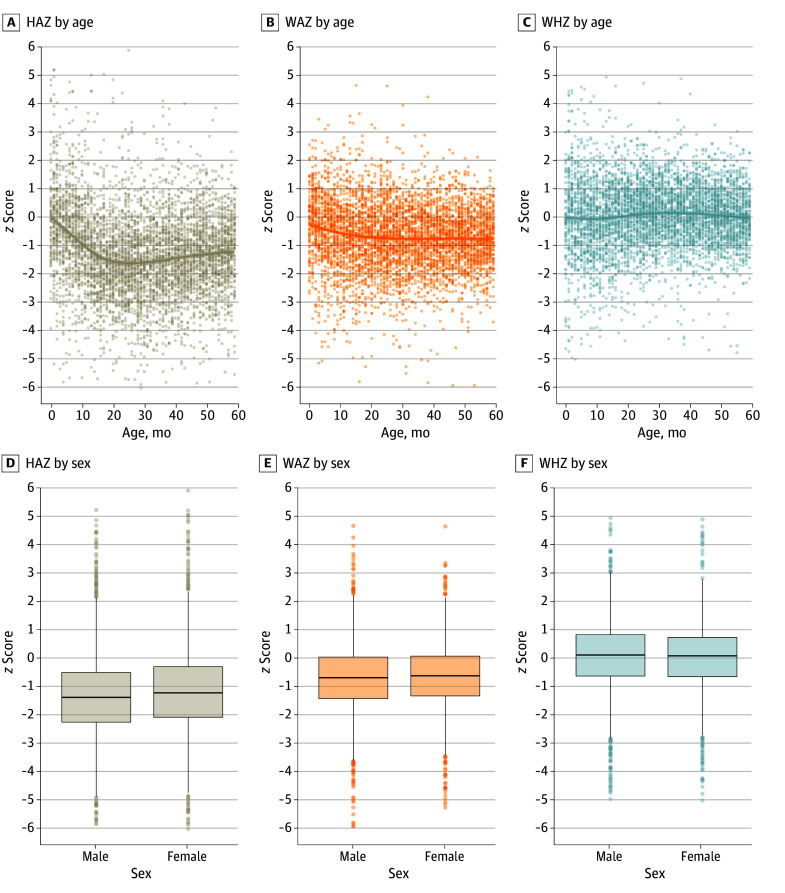
Distribution of Anthropometric *z* Scores as a Function of Age and Sex A-C, Data were fitted by thin plate regression spline. Growth faltering is seen according to the HAZ. Mean HAZ started below the 2006 World Health Organization Child Growth Standards, with *z* score deficits ranging from −0.5 at 6 months to −1.5 at 18 months. Dots represent individuals. D-F, The horizontal bar in each box plot represents the median value for the *z* scores; edges of the boxes, the first and third quartiles; width of the boxes, IQRs. Whiskers extend to the smallest and largest observations within 1.5 times the IQR of the quartiles. Dots represent outliers. HAZ indicates height-for-age *z* score; WAZ, weight-for-age *z* score; WHZ, weight-for-height *z* score.

**Figure 2.**
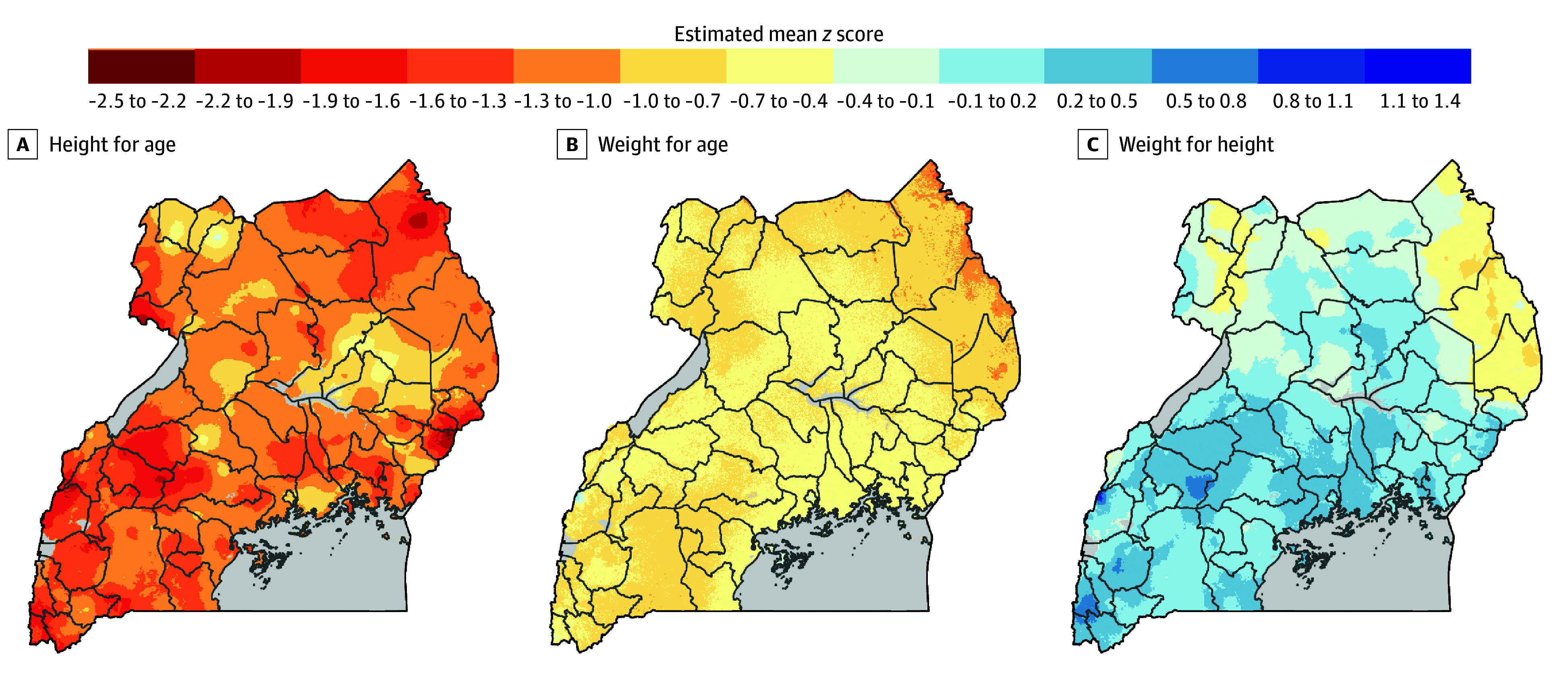
Spatial Distribution of Anthropometric *z* Scores for Individuals Younger Than 5 Years in Uganda The *z* scores were derived using the 2006 World Health Organization Child Growth Standards.

Of the 5219 individuals aged 0 to 59 months included in the analysis, 2633 (50%) were female and 2586 (50%) were male; mean (SD) age was 29 (17) months. Most individuals (4352 [83%]) resided in rural areas, and 2471 (47%) lived in households with the lowest and second-lowest wealth quintiles (eTable 2 in Supplement 1). The median HAZ declined sharply from birth until 20 months of life and then increased gradually until age 5 years but remained below 0. The median WAZ remained below 0 throughout the 5 years without signs of recovery. The median WHZ did not demonstrate a consistent pattern across the 5 years, hovering around the normal median *z* score of 0 (Figure 1A-C). The mean (SD) HAZ was −1.23 (1.48), mean (SD) WAZ was 0.65 (1.13), and mean (SD) WHZ was 0.08 (1.15). Overall, the prevalence of stunting was 30.22% (95% CI, 29.36%-30.98%), of underweight was 12.23% (95% CI, 11.55%-12.91%), and of wasting was 3.63% (95% CI, 3.46%-3.80%). Sex-specific medians of anthropometric measures are shown in Figure 1D-F. Spatial heterogeneity in HAZ, WAZ, and WHZ was observed in Uganda (Figure 2).

### Optimal Geostatistical Model

The model estimates for HAZ, WAZ, and WHZ reported in the Table are the results of a model selection procedure based on the Akaike information criterion (AIC). Exploration of the models with lags revealed that the lowest AIC was achieved with a model with lags *D* = 8 months for EVI, 11 months for rainfall, and 3 months for SPEI (Table). Higher SPEI at 3 months before birth was positively associated with childhood growth outcomes (Table); the effect size (β) was 0.06 (95% CI, 0.02-0.10) for HAZ, 0.04 (95% CI, 0.01-0.07) for WAZ, and 0.03 (95% CI, 0.001-0.06) for WHZ. Additionally, higher location mean rainfall at 11 months before birth (β, 0.06; 95% CI, 0.01-0.10) and higher land-surface mean temperature (β, 0.10; 95% CI 0.01-0.19) were positively associated with HAZ, consistent with SPEI findings. Associations of the Aridity Index with WAZ (β, 0.09; 95% CI, 0.04-0.13) and WHZ (β, 0.09; 95% CI, 0.02-0.16) were consistent with findings for SPEI. EVI and slope angle were not consistently associated with growth metrics. Travel time to the nearest city and nighttime light emissions also were not associated with growth outcomes.

**Table.** Maximum Likelihood Estimates and Corresponding 95% CIs for the Model

Parameter^a^	HAZ, β (95% CI)^b^	*P* value	WAZ, β (95% CI)^b^	*P* value	WHZ, β (95% CI)^b^	*P* value
Intercept	−1.23 (−1.32 to −1.14)	NA	−0.64 (−0.68 to −0.60)	NA	0.04 (−0.07 to 0.15)	NA
Slope angle	−0.03 (−0.10 to 0.04)	.37	−0.06 (−0.11 to −0.01)	.02	−0.009 (−0.06 to 0.04)	.74
Land-surface mean temperature	0.10 (0.01 to 0.19)	.04	0.004 (−0.05 to 0.06)	.88	−0.05 (−0.15 to 0.05)	.32
Aridity Index	−0.002 (−0.08 to 0.07)	.97	0.09 (0.04 to 0.13)	<.001	0.09 (0.02 to 0.16)	.008
Travel time to nearest city	−0.01 (−0.07 to 0.05)	.77	−0.02 (−0.06 to 0.02)	.28	−0.03 (−0.08 to 0.02)	.19
Nighttime light emissions	0.02 (−0.06 to 0.10)	.59	0.03 (−0.01 to 0.07)	.18	−0.04 (−0.09 to 0.01)	.15
EVI 8 mo before birth	−0.05 (−0.10 to −0.004)	.03	0.02 (−0.02 to 0.05)	.37	0.04 (0.003 to 0.08)	.03
Mean rainfall 11 mo before birth	0.06 (0.01 to 0.10)	.009	0.03 (−0.001 to 0.06)	.06	0.02 (−0.01 to 0.06)	.14
SPEI 3 mo before birth	0.06 (0.02 to 0.10)	.005	0.04 (0.01 to 0.07)	.009	0.03 (0.001 to 0.06)	.04
Between-cluster variation, σ^2^^c^	0.15 (0.11 to 0.21)	NA	0.11 (0.08 to 0.14)	NA	0.08 (0.05 to 0.12)	NA
Range parameter ϕ, km^d^	18.28 (9.78 to 34.17)	NA	NA	NA	32.17 (9.14 to 113.24)	NA
Residual variation, ω^2^^e^	2.02 (1.01 to 4.05)	NA	1.27 (0.10 to 16.73)	NA	1.26 (0.50 to 3.17)	NA

Abbreviations: EVI, Enhanced Vegetation Index; HAZ, height-for-age *z* score; NA, not applicable; SPEI, Standardized Precipitation-Evapotranspiration Index; WAZ, weight-for-age *z* score; WHZ, weight-for-height *z* score.

^a^ Slope angle, land surface temperature, Aridity Index, travel time to nearest city, and nighttime light emissions are temporally constant variables. EVI, rainfall, and SPEI are temporally varying variables.

^b^ For continuous climatic or weather variables, an increase of 1 SD results in a β increase in the mean HAZ, WAZ, and WHZ.

^c^ Between-cluster variation reflects the spatial variation in the anthropometric measurements.

^d^ The scale parameter (ϕ) controls the rate of decay of the correlation between observations as distance increases. A larger ϕ value indicates that spatial correlation persists over a longer distance.

^e^ Indicates the individual-specific (nonspatial) residual variation in anthropometric measurements.

The individual-specific components of the residual variation (ω^2^) for HAZ (2.02; 95% CI, 1.01-4.05), WAZ (1.27; 95% CI, 0.10-16.73), and WHZ (1.26; 95% CI, 0.50-3.17) were larger than the between-cluster variations (σ^2^) (HAZ: 0.15 [95% CI, 0.11-0.21]; WAZ: 0.11 [95% CI, 0.08-0.14]; WHZ: 0.08 [95% CI, 0.05-0.12]). The range parameter (ϕ) for WHZ (32.17 km; 95% CI, 9.14-113.24 km) was larger than that for HAZ (18.28 km; 95% CI, 9.78-34.17 km), suggesting that spatial correlation persisted over a longer range for this malnutrition indicator. Therefore, the practical range of the spatial correlation for WHZ and HAZ was 96.51 km and 54.84 km, respectively. The ϕ parameter for WAZ was not identifiable due to the absence of residual spatial variation (or it was at such a small scale that we did not have enough data locations to identify it).

### Precision Spatial Estimation of Childhood Growth Faltering

**Figure 3.**
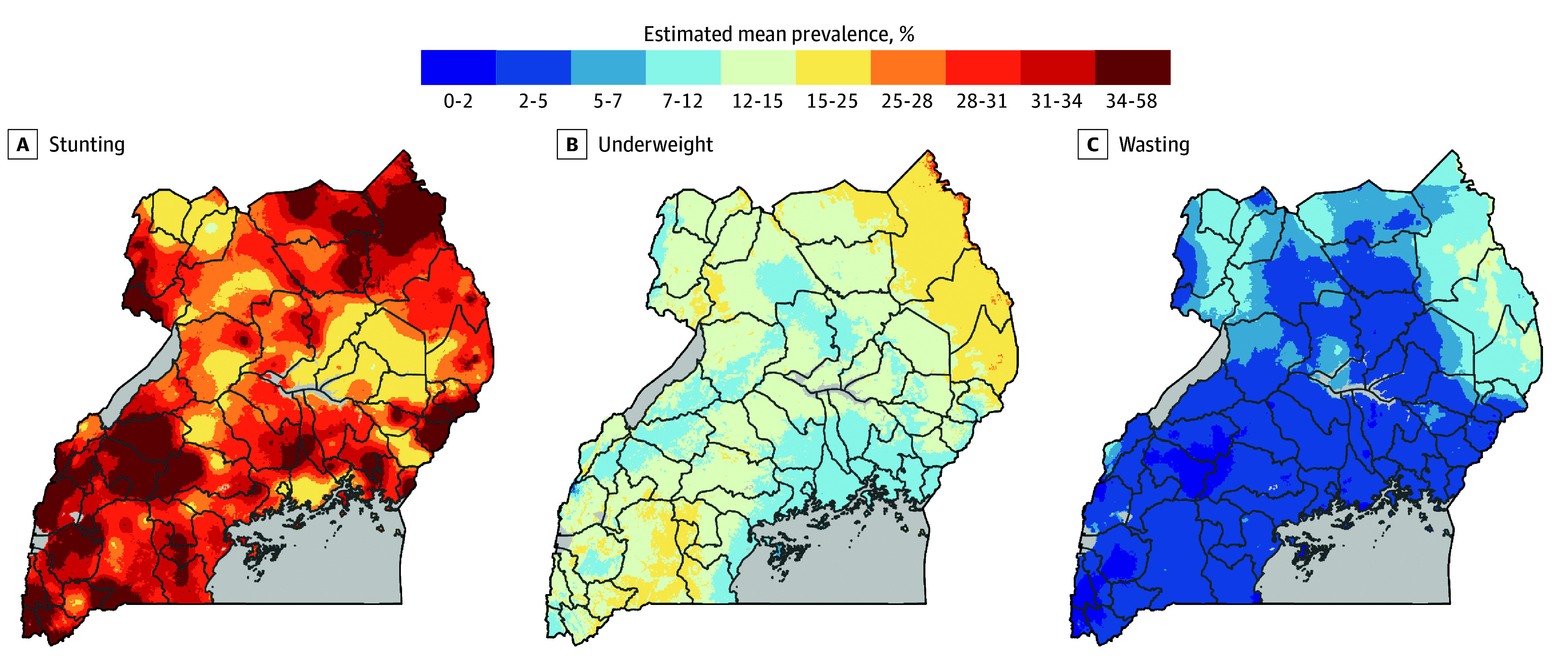
Spatial Variation of Undernutrition Prevalence in Uganda A, Assessed as height-for-age *z* score. B, Assessed as weight-for-age *z* score. C, Assessed as weight-for-height *z* score.

The data showed stunting to be the most prevalent form of childhood growth faltering in Uganda. Subnational spatial heterogeneity was found to exist in Uganda, with the northeastern and western regions having the highest mean prevalence of stunting at more than 40% (>10 percentage points higher than the national mean prevalence of 30.22% [95% CI, 29.36%-30.98%]). Distinct regional areas with high prevalence were distributed along the Lake Albert, Lake Edward, and Lake George banks in the southwestern region near the Democratic Republic of the Congo, in addition to clusters in the far northeast (Figure 3A). On average, spatial clusters of regional stunting were 55-km wide.

The national estimated prevalence of underweight was 12.23% (95% CI, 11.55%-12.91%), although rates higher than 16% were found in areas in the northeastern regions of Karamoja, Moroto, Kotido, and Mt Elgon at the border of the Rift Valley Province of Kenya (Figure 3B). Smaller areas with high prevalence were observed in the southwestern region of Mbarara bordering Tanzania and Rwanda and in the Kasese region surrounding the slopes of the Rwenzori Mountains.

The national prevalence of wasting was 3.63% (95% CI, 3.46%-3.80%). Spatial clusters of wasting were evident in northeastern and northwestern regions, with prevalence greater than 6% (Figure 3C). Northeastern regions included Karamoja, Moroto, Kotido, and Mt Elgon at the border of the Rift Valley Province of Kenya. Northwestern regions were in Arua and Nebbi along the banks of the Albertine Nile. On average, these clusters were 97-km wide.

**Figure 4.**
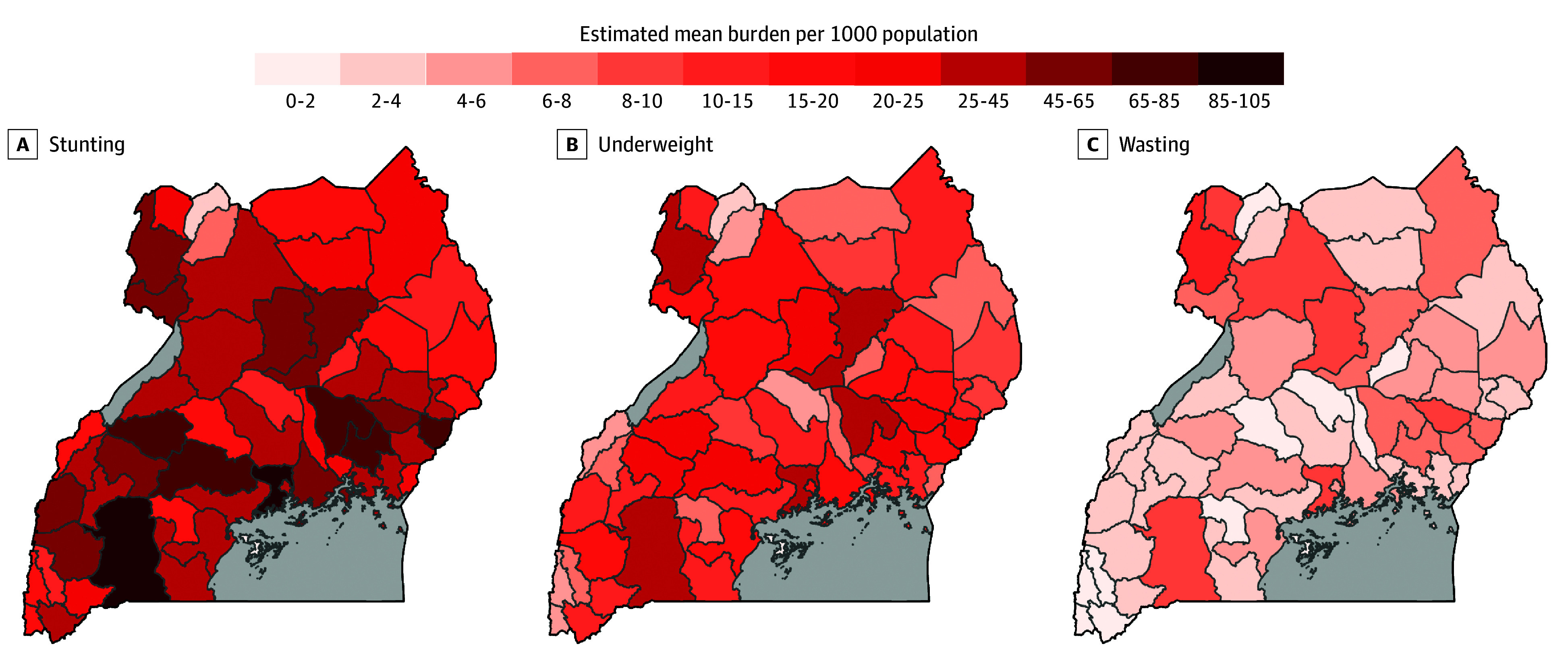
Burden of Childhood Undernutrition in Uganda Absolute numbers at the district level were derived using the 2020 population younger than 5 years, with the assumption that prevalence of undernutrition did not change between 2016 and 2020. A, Assessed as height-for-age *z* score. B, Assessed as weight-for-age *z* score. C, Assessed as weight-for-height *z* score.

In 2020, of 7.2 million individuals younger than 5 years in Uganda, 2.2 million (30.56%) had stunting. Geographic locations with the largest population younger than 5 years had the highest absolute numbers of individuals with growth faltering (Figure 4).

## Discussion

In this study, we found that childhood growth faltering was a severe public health problem in Uganda, with subnational prevalence estimates of stunting, underweight, and wasting often exceeding 40%, 16% and 6%, respectively. We estimated that 2.2 million (of 7.2 million) individuals younger than 5 years had stunting in 2020.

SPEI, rainfall, and the Aridity Index were associated with nutritional outcomes before conception and during pregnancy, supporting previous findings that public health interventions to improve women’s nutrition before and during pregnancy could positively impact the offspring’s nutritional outcome at birth and later in life.^37^ We expected EVI to be associated with better growth outcomes, but this index demonstrated inconsistent associations across growth metrics. Additionally, we expected areas of higher poverty (as identified by lower nighttime light emission) to be associated with poor growth outcomes, but no such association was found. Slope angle and temperature demonstrated inconsistent associations with growth outcomes.

### Strengths and Limitations

The present results have several notable strengths and fill critical knowledge gaps. First, our findings extend the recent demonstration of growth faltering at birth and the associations of rainfall with wasting following birth^3,4,5^ to environmental factors before birth. Second, our fine-scale risk maps can be used as operational tools to inform precision intervention. Third, unlike previous undernutrition analyses in which prediction models were based on binary anthropometric measurements,^38^ our parameter estimation and spatial estimation were fitted on continuous anthropometric measurements before creating prevalence risk maps. In the context of geostatistical inference, dichotomization of continuous outcomes can lead to a substantial loss of efficiency for both parameter estimation and spatial estimation. It also can result in the loss of fine-scale features of disease prevalence.^33^ Fourth, our currently generated childhood growth faltering risk maps showed anthropometric spatial variations, which may be affected by the ancestry and genetic variations of the Ugandan population. Uganda has a diverse ethnolinguistic composition (>52 tribal associations). We observed that some of the clusters in stunting maps followed geographic ethnic distributions. We are working on a large-scale ancestral genomic analysis of Ugandan people.

Our findings should be viewed in light of the study’s assumptions and limitations. First, childhood growth faltering is more complex than the factors examined herein and is associated with childhood nutrition after birth, access to and quality of health care, overall level of sanitation, maternal educational level, level of poverty, and access to safe drinking water.^39^ Second, remotely sensed environmental variables were used as proxies for agricultural yield, yet in the absence of nutritional intake data using validated food frequency instruments, the relationship with crops remains uncertain. Third, agricultural yields in response to rains are confounded by the relationship of rainy seasons with infectious disease burden. Fourth, an important concern in any study of growth faltering among African children younger than 5 years using the 2006 WHO Child Growth Standards as a reference is that individuals from only 1 African country (Ghana, West Africa) were represented. Such an undersampling of the African population in the current normative growth curves introduces bias in the estimation of faltering in childhood growth in diverse African peoples as represented within Uganda.

## Conclusions

In this cross-sectional study, we developed precision maps displaying spatial variation of childhood growth faltering among individuals younger than 5 years in Uganda and estimated its association with environmental factors prior to birth. The findings of this study demonstrated that prenatal and preconceptual environmental factors of rainfall and water availability were associated with long-term childhood growth outcomes.

## Notes

Supplement 1. **eTable 1.** Sources for Data Included in the Model **eTable 2.** Demographic Characteristics of the Study Population **eFigure 1.** Temporally Changing Standardized Precipitation-Evapotranspiration Index **eFigure 2.** Correlation Between SPEI and EVI **eFigure 3.** Temporally Changing Precipitation **eFigure 4.** Land Surface Mean Temperature **eFigure 5.** Correlation Between EVI and NDVI **eFigure 6.** Temporally Changing EVI **eFigure 7.** Aridity Index **eFigure 8.** Nighttime Light Emissions **eFigure 9.** Drive Time to the Nearest City **eFigure 10.** Correlation Matrix of Environmental or Weather Factors **eFigure 11.** Slope Angle

Supplement 2.
Data Sharing Statement
