# Supplementary material for: Preconception and Prenatal Environment and Growth Faltering Among Children in Uganda
Source: JAMA Netw Open. 2025 Mar 19;8(3):e251122. doi: 10.1001/jamanetworkopen.2025.1122 (PMC11923699; doi:10.1001/jamanetworkopen.2025.1122)
Supplement: Supplement 1. — eTable 1. Sources for Data Included in the Model eTable 2. Demographic Characteristics of the Study Population eFigure 1. Temporally Changing Standardized Precipitation-Evapotranspiration Index eFigure 2. Correlation Between SPEI and EVI eFigure 3. Temporally Changing Precipitation eFigure 4. Land Surface Mean Temperature eFigure 5. Correlation Between EVI and NDVI eFigure 6. Temporally Changing EVI eFigure 7. Aridity Index eFigure 8. Nighttime Light Emissions eFigure 9. Drive Time to the Nearest City eFigure 10. Correlation Matrix of Environmental or Weather Factors eFigure 11. Slope Angle [file jamanetwopen-e251122-s001.pdf]

Supplementary Online Content

Ssentongo P, Fronterre C, Ericson JE, et al. Preconception and prenatal environment and growth faltering among children in Uganda. *JAMA Netw Open*. 2025;8(3):e251122. doi:10.1001/jamanetworkopen.2025.1122

eTable 1. Sources for Data Included in the Model
eTable 2. Demographic Characteristics of the Study Population
eFigure 1. Temporally Changing Standardized Precipitation-Evapotranspiration Index
eFigure 2. Correlation Between SPEI and EVI
eFigure 3. Temporally Changing Precipitation
eFigure 4. Land Surface Mean Temperature
eFigure 5. Correlation Between EVI and NDVI
eFigure 6. Temporally Changing EVI
eFigure 7. Aridity Index
eFigure 8. Nighttime Light Emissions
eFigure 9. Drive Time to the Nearest City
eFigure 10. Correlation Matrix of Environmental or Weather Factors
eFigure 11. Slope Angle

This supplementary material has been provided by the authors to give readers additional information about their work.

© 2025 Ssentongo P et al. *JAMA Network Open*.

Variable	Category	Source	Web links	Comment
**Anthropometric measures**	Nutrition	Demographic and Health Survey (DHS)	DHS Website	
**Population, 2020, UN adjusted**	Demography	WorldPop, Population count	WorldPop Website.	Documentation
**Shapefiles**	Geography	GADM Maps and Data	GADM Website.	Documentation
**Precipitation**	Meteorology	National Oceanic and Atmospheric Administration (NOAA) RFE2 product	Climate Prediction Center Website	Documentation.
**Mean temperature**	Meteorology	Climatic Research Unit (CRU)	CRU Website.	Documentation
**SPEI**	Meteorology	National Center for Atmospheric Research/University Corporation Atmospheric Research (UCAR/NCAR)	UCAR/NCAR Website	Documentation
**Aridity index**	Meteorology	CGIAR Consortium for Spatial Information (CGIAR-CSI)		Documentation
**Elevation**	Topography	Elevation above the sea level (in meters) based on NASA's Shuttle RadarTopography Mission (SRTM) |	WorldPop Website	Documentation.
**Slope angle**	Topography	Topographic slope (in degree) based on NASA's Shuttle Radar TopographyMission (SRTM)	WorldPop Website	Documentation
**EVI and NDVI**	Vegetation cover	National Aeronautics and Space Administration (NASA)	NASA website	Documentation
**Nighttimelights**	Economic development indicator	Geospatial covariate, night-time lights in Uganda	WorldPopWebsite	Documentation
**Distance to major road**	Economic development indicator	Distance to nearest major road in Uganda	WorldPop Website	
**Travel time to city**	Economic development indicator	Travel time to city (minutes) in Uganda	WorldPop Website	Documentation

eTable 1. Sources for data included in the model. Current versions of this data can be found at the links included. SPEI: Standardized precipitation-evapotranspiration index. SPEI is rainfall minus potential evaporation and transpiration by plants describes the water balance, with negative values indicating drought potential. Index is normalized, and thus express deviations from the local long-term climatology. Enhanced vegetation index (EVI) and normalized difference vegetation index (NDVI) are satellite derived vegetation indexes; smaller values indicate less vegetation. SPEI has a multi-scale character, providing SPEI timescales between 1 and 48 months. We analyzed a 2-month lag SPEI. Aridity index is given as the ratio of mean annual precipitation to mean annual potential evapotranspiration. Values closer to zero indicate arid climates. Dataset use values from 1970-2000 period.

© 2025 Ssentongo P et al. *JAMA Network Open*.

Variable (N=5219)	Mean (SD)
**Child factors**	
Age, month	29 ± 17
Male sex*	2586 (50)
Height-for-age z scores	-1.23 ±1.48
Stunted*	1529 (29)
Weight-for-height z-scores	0.08 ± 1.15
Wasted*	189 (4)
Weight-for-age z-scores	-0.65 ± 1.13
Underweight*	570 (11)
**Household factors**	
Wealth index quintiles*	
Lowest	1359 (26)
Second	1112 (21)
Middle	1047 (20)
Fourth	950 (18)
Highest	751(14)
Rural residence*	4352 (83)
**Maternal Factors**	
Maternal education*	
Less than high school	490 (67)
High School	839 (16)
Greater than high school	242 (5)
Missing	648 (12)

*n (%)

eTable 2. Demographic characteristics of the study population. Child, household, and maternal-specific demographic characteristic for the study population.

© 2025 Ssentongo P et al. *JAMA Network Open*.

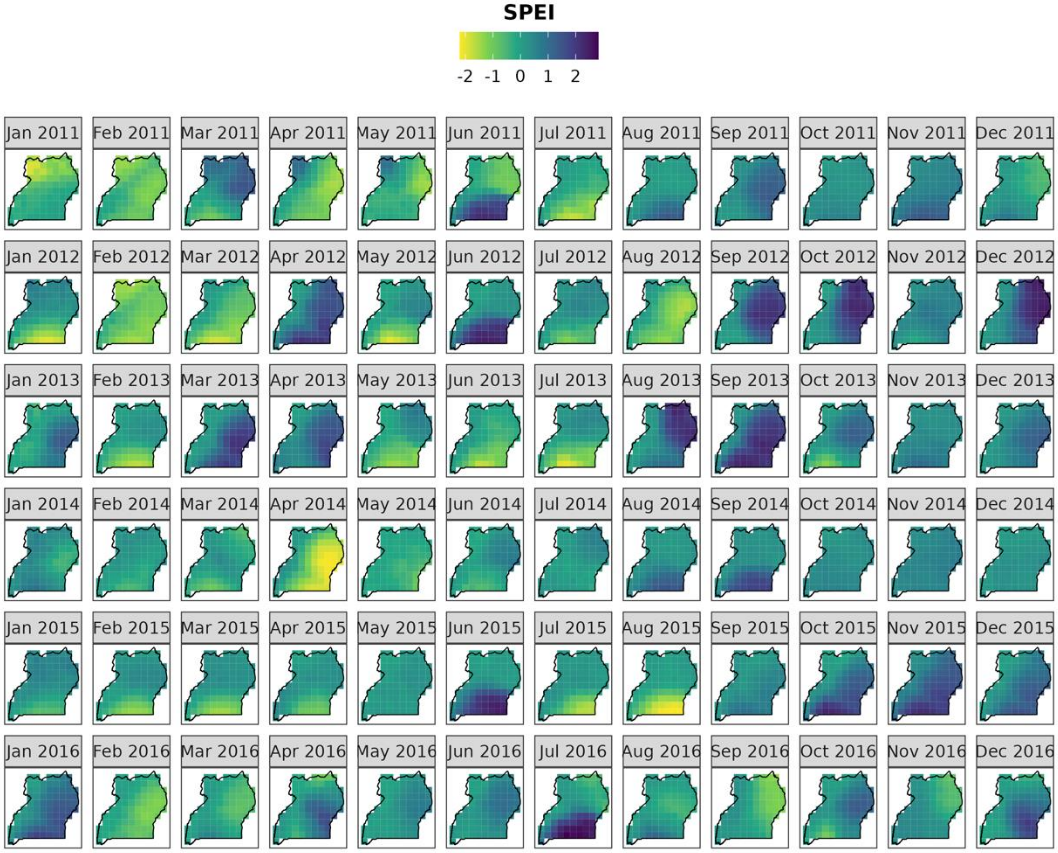

eFigure 1: Temporally changing standardized precipitation-evapotranspiration index (SPEI): Monthly measure of climatic water balance between January 2011 to December 2016.

© 2025 Ssentongo P et al. *JAMA Network Open*.

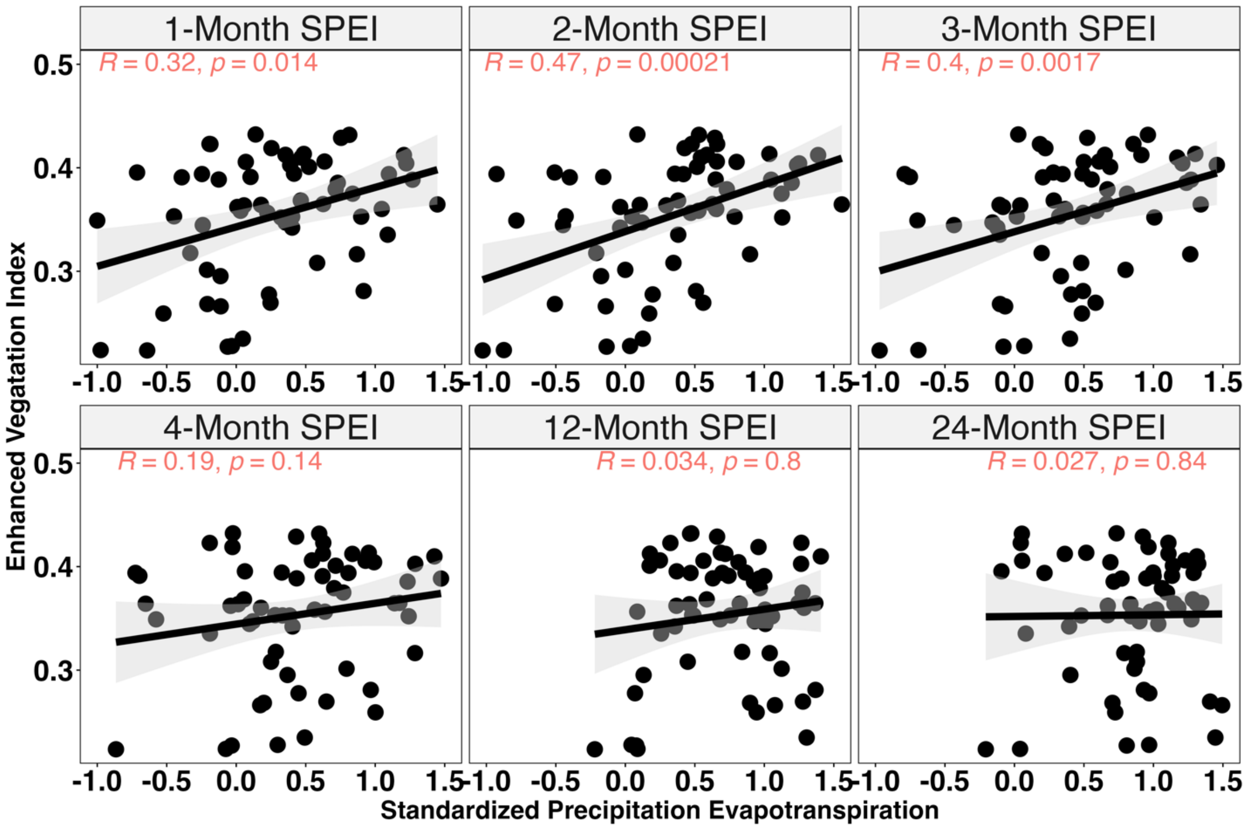

eFigure 2: Correlation between SPEI and EVI. EVI was correlated (Pearson coefficient) to the monthly 1- to 4-mo, 12-mo and 24-mo SPEI series. A 2-mo SPEI (with the highest Pearson correlation coefficient) was incorporated in our modeling.

© 2025 Ssentongo P et al. *JAMA Network Open*.

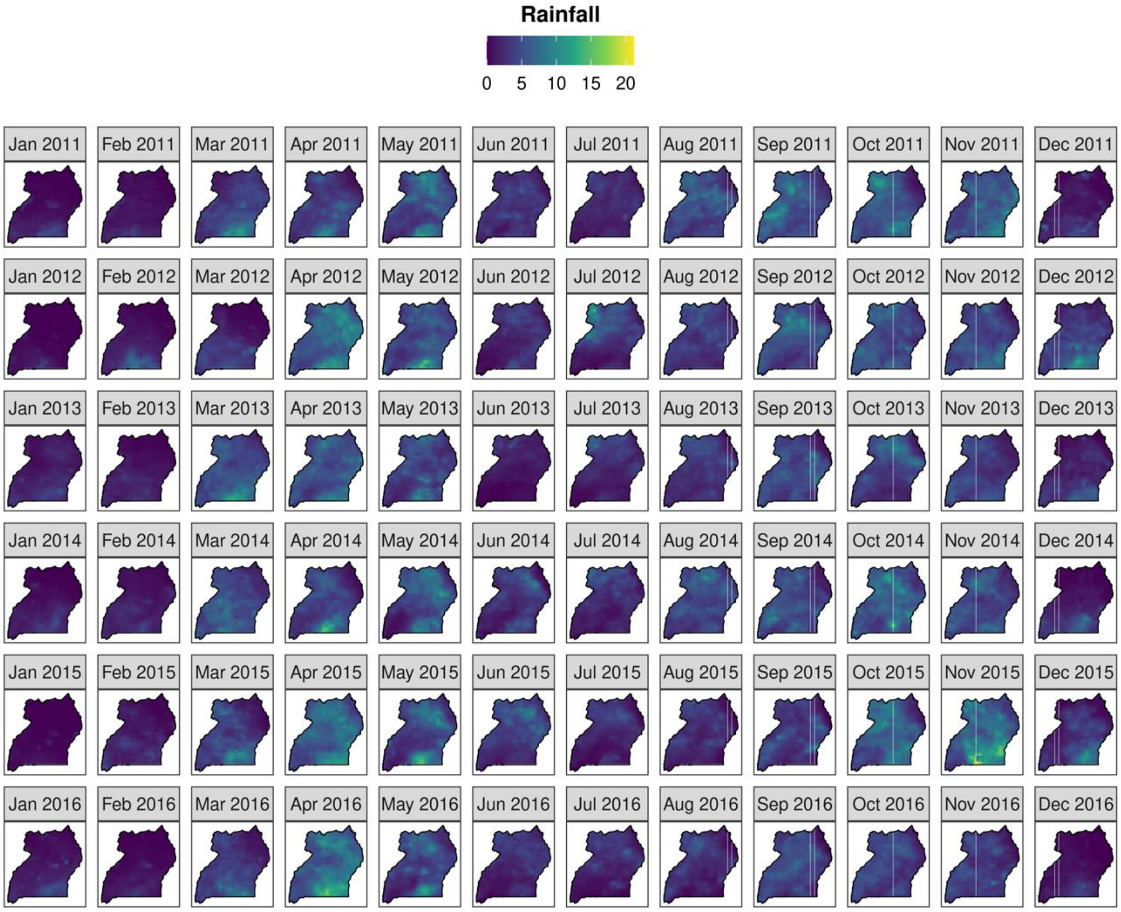

eFigure 3: Temporally changing rainfall. Monthly mean rainfall between January 2011 to December 2016. Spatial resolution of 1-km grid square.

© 2025 Ssentongo P et al. *JAMA Network Open*.

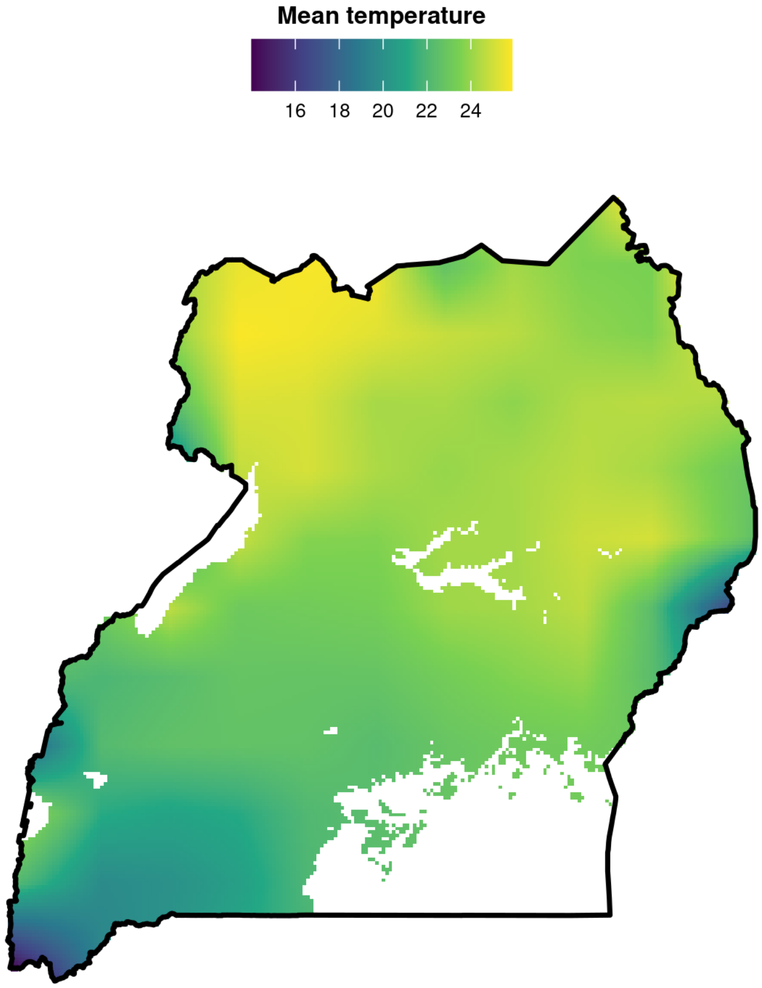

eFigure 4. Land surface mean temperature. Mean annual temperature in degrees Celsius.

© 2025 Ssentongo P et al. *JAMA Network Open*.

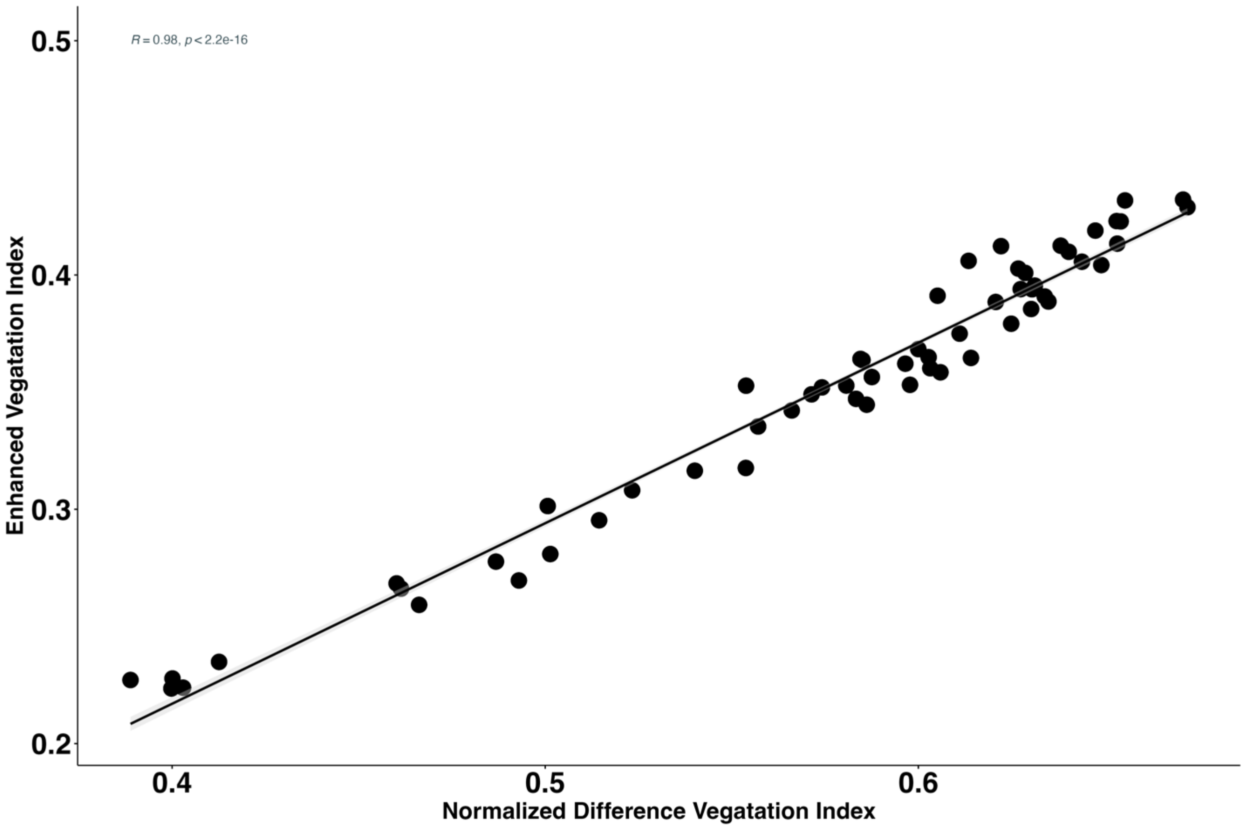

eFigure 5. Correlation between EVI and NDVI. Pearson correlation coefficient between NDVI and EVI was 98%. We included EVI in our model.

© 2025 Ssentongo P et al. *JAMA Network Open*.

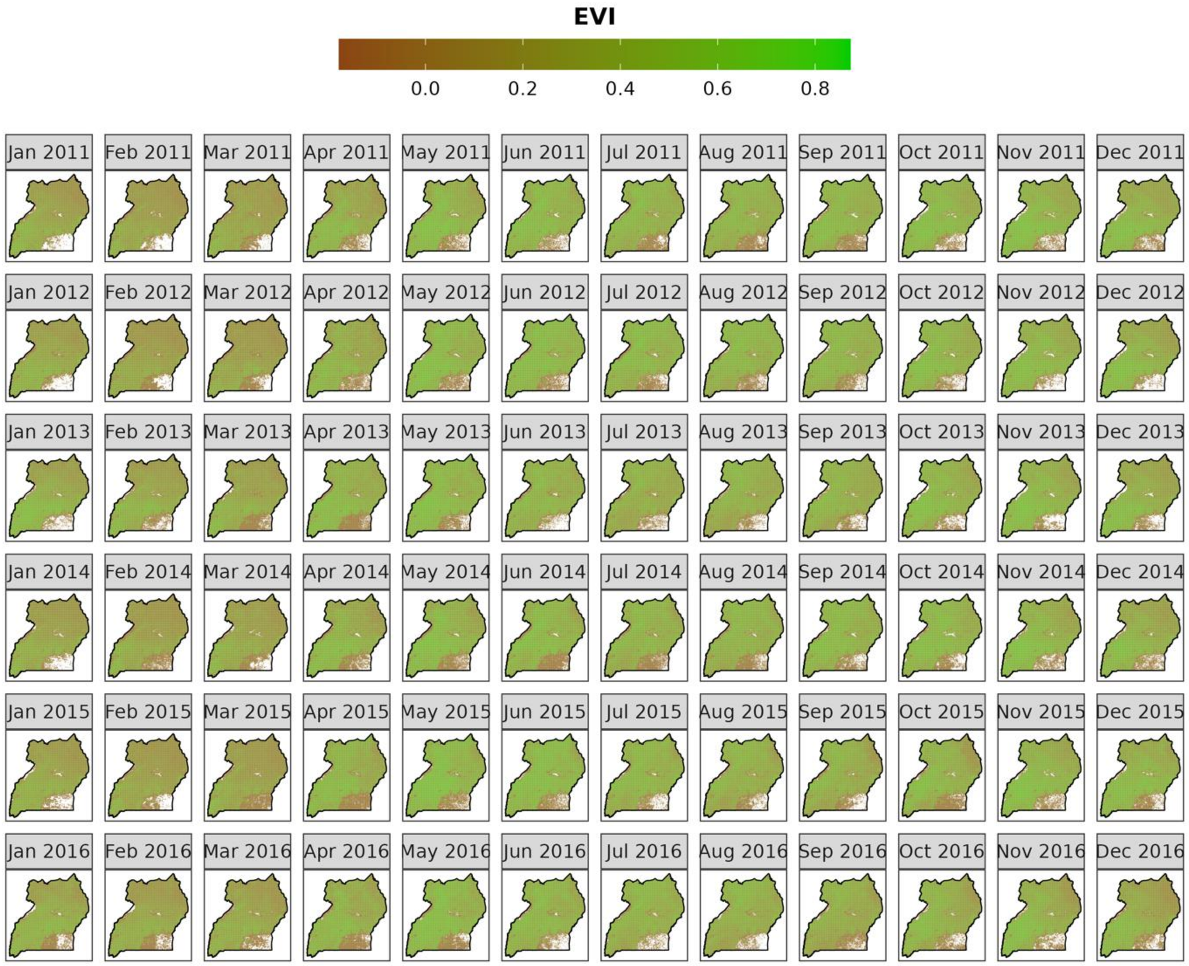

eFigure 6: Temporally changing EVI. Monthly EVI between January 2011 to December 2016. Spatial resolution of 1-km grid square.

© 2025 Ssentongo P et al. *JAMA Network Open*.

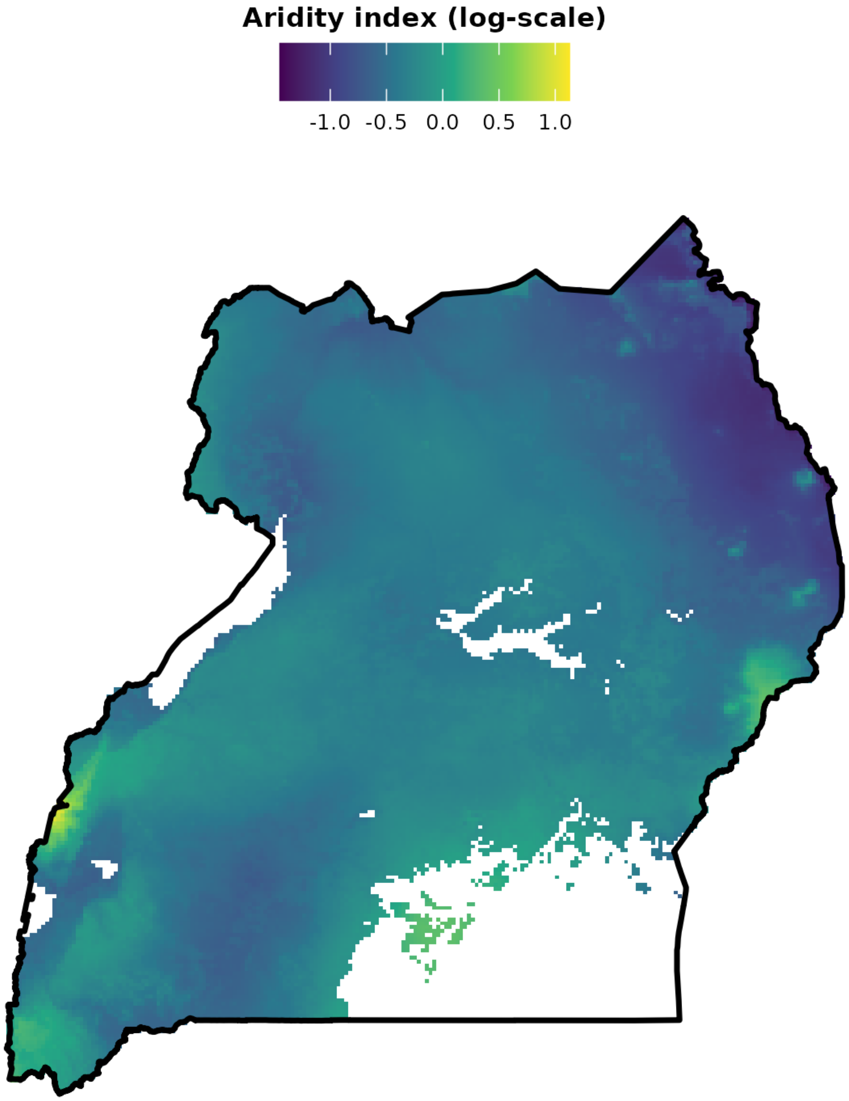

eFigure 7. Aridity Index. Higher values depict humid regions and lower values depict arid regions.

© 2025 Ssentongo P et al. *JAMA Network Open*.

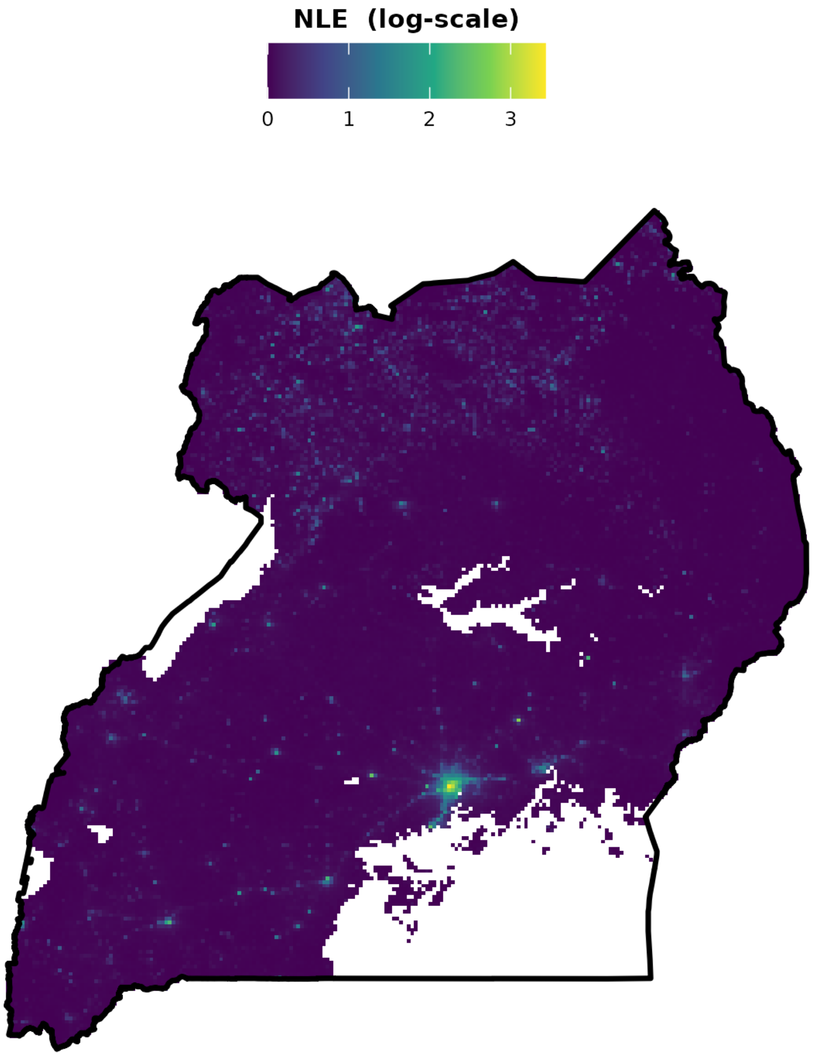

eFigure 8. Nighttime light emissions.

© 2025 Ssentongo P et al. *JAMA Network Open*.

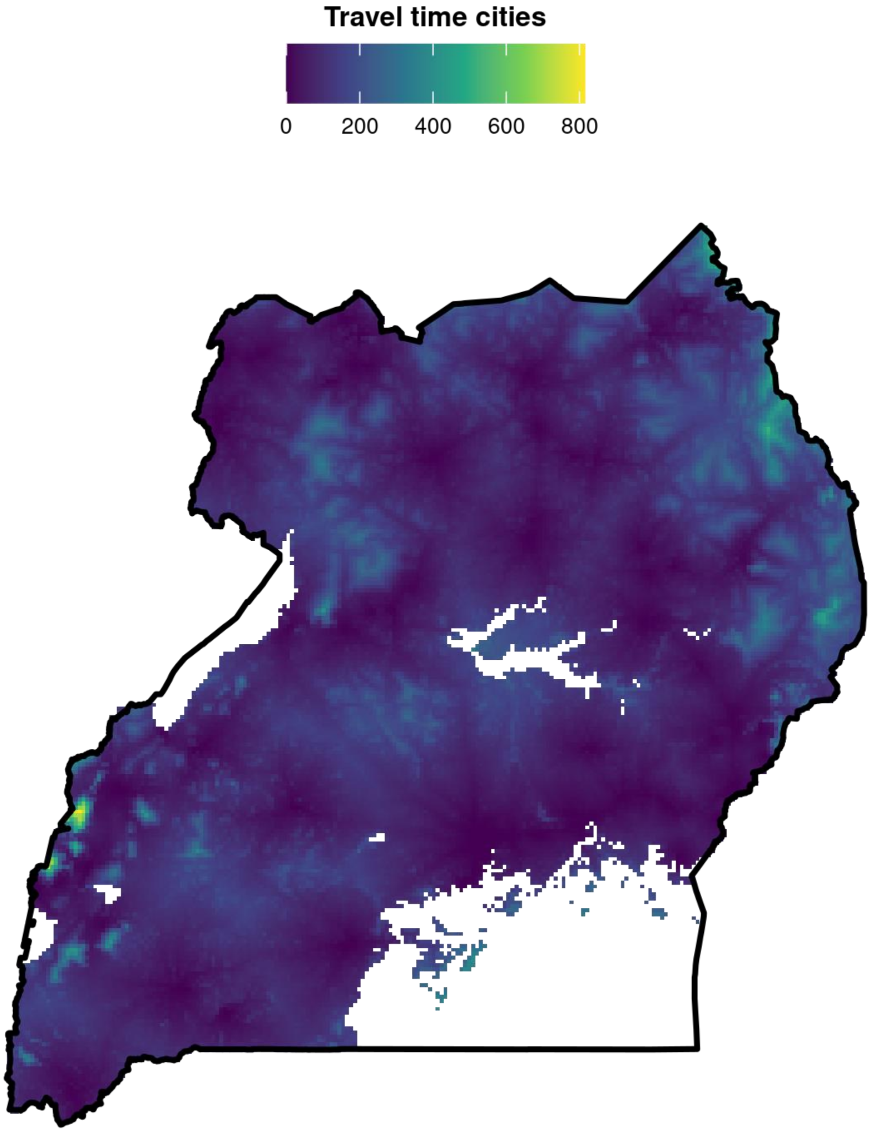

eFigure 9. Drive time to the nearest city. Travel time (hours) to nearest city.

© 2025 Ssentongo P et al. *JAMA Network Open*.

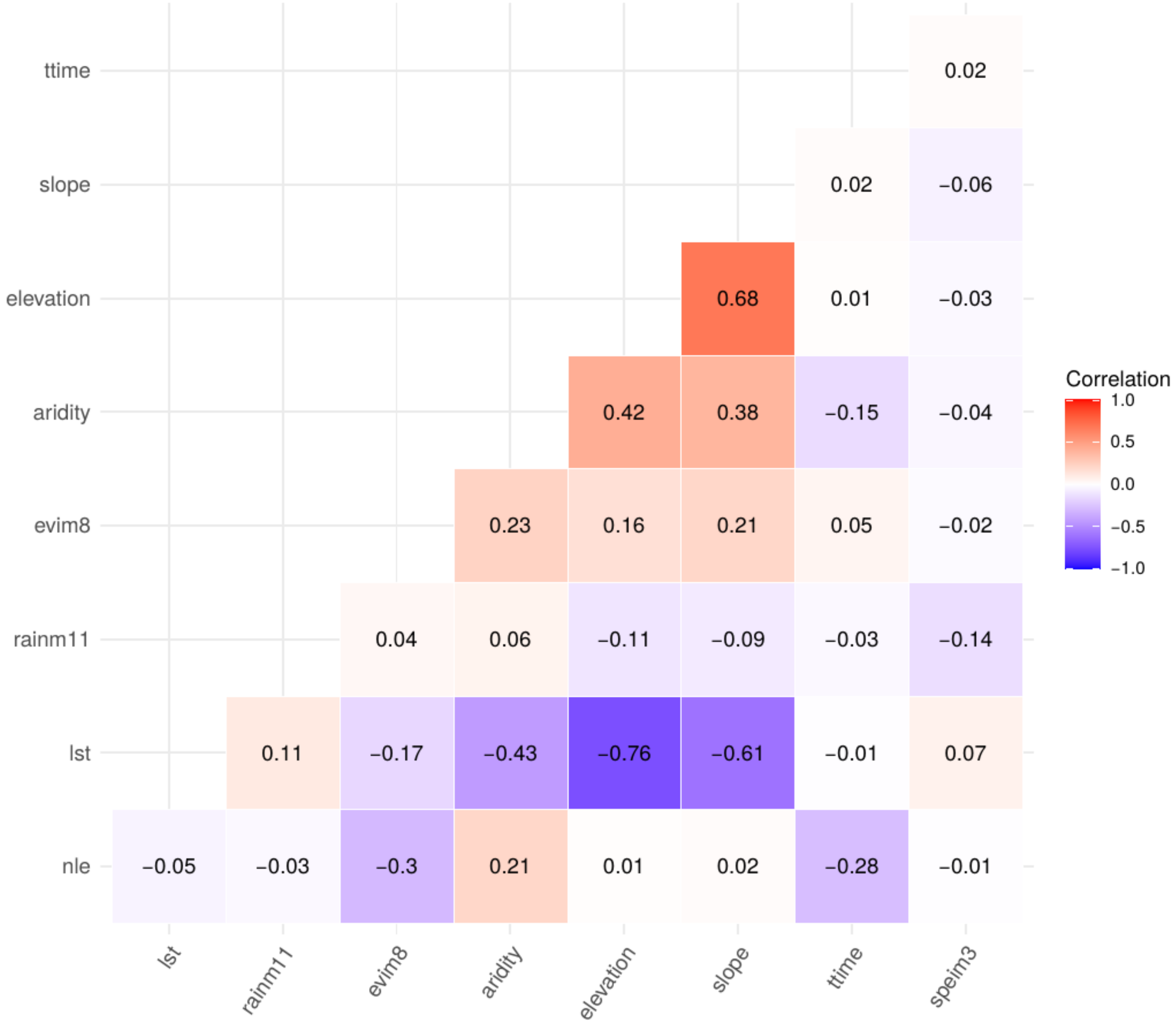

eFigure 10. Correlation matrix of environmental/weather factors. lst: land surface temperature (Monthly mean); rainm11: Rainfall 11 months before birth; evim8: EVI 8 months before birth; slope: Slope angle; nle: Nighttime light emission; ttime: Travel time to city; speim3: SPEI three months before birth.

© 2025 Ssentongo P et al. *JAMA Network Open*.

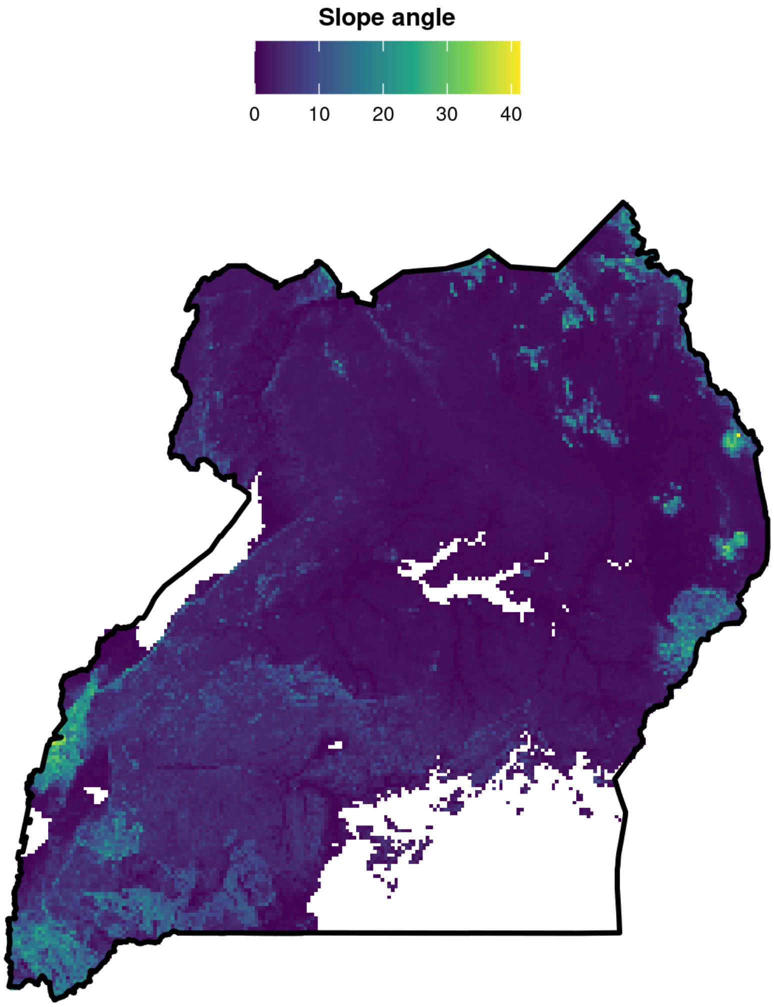

eFigure 11. Slope angle. Slope angle in degrees.

© 2025 Ssentongo P et al. *JAMA Network Open*.
