# Supplementary material for: Preconception and Prenatal Environment and Growth Faltering Among Children in Uganda
Source: JAMA Netw Open. 2025 Mar 19;8(3):e251122. doi: 10.1001/jamanetworkopen.2025.1122 (PMC11923699; doi:10.1001/jamanetworkopen.2025.1122)
Supplement: Supplement 2. — Data Sharing Statement [file jamanetwopen-e251122-s002.pdf]

Data Sharing Statement

Ssentongo. Preconception and Prenatal Environment and Growth Faltering Among Children in Uganda. *JAMA Netw Open*. Published March 19, 2025.
doi:10.1001/jamanetworkopen.2025.1122

Data
Data available: Yes
Data types: Deidentified participant data
How to access data: Provided data and R code to reproduce the results. These can be found on GitHub
When available: With publication

Supporting Documents
Document types: Statistical/analytic code
How to access documents: R code and data to reproduce the results in this manuscript are archived at GitHub (https://github.com/claudiofronterre/malnutrition_uganda). The predictive maps produced in this paper can be explored interactively through the following Shiny app https://claudiof.shinyapps.io/undernutrition_uganda/.
When available: With publication

Additional Information
Who can access the data: anyone requesting the data
Types of analyses: academic/ research purpose
Mechanisms of data availability: investigator support
